# Augmented-reality enhanced ultrasound guidance: a sterile, hands-free approach using commercial augmented reality headsets

**DOI:** 10.1007/s12194-026-01035-z

**Published:** 2026-03-04

**Authors:** Thomas Saliba, David Rotzinger, Giuseppe Gullo

**Affiliations:** 1 Radiology department, Centre Hospitalier Universitaire Vaudois (CHUV), Rue du Bugnon 46, Lausanne, 1005 Switzerland; 2 Université de Lausanne, Lausanne, Switzerland

**Keywords:** Augmented reality, Virtual Reality, Mixed Reality, Ultrasound, Radiology, POCUS

## Abstract

We present a cost-effective method for using commercially available augmented reality (AR) headsets to enable sterile, hands-free ultrasound control. A Meta Quest 3 headset was paired with portable ultrasound probes via USB-C, with the Philips Lumify app sideloaded onto the headset for hand-tracking interaction. This allowed real-time imaging, screen resizing, and repositioning while maintaining sterility, even with surgical gloves. Using the Lumify app AR all of the settings and functionalities, such as gain, depth and Doppler, can be modified as they would be if using the app on a tablet. Image quality matched that of tablets, there was no glare, and remote collaboration was supported. This method requires no hardware beyond an AR headset which runs android and is capable of sideloading applications and an ultrasound probe with an accompanying android app. This proof-of-concept highlights potential for AR-enhanced ultrasound in procedural guidance and teleradiology, offering improved sterility and accessibility.

**Supplementary Information:**

The online version contains supplementary material available at https://doi.org/10.1007/s12194-026-01035-z.

## Introduction

Virtual reality (VR) and augmented reality’s (AR) use in radiology has become increasingly popular as the technology has matured, finding a wide variety of use cases ranging from preparing patients for MRI exams to rehabilitation [1–3]. 

Using AR technology to augment the use of ultrasound has been considered as far back as 1996, with researchers using it to display images on a head mounted system to facilitate percutaneous breast biopsies using needles [4]. 

In some cases, seeing the screen superimposed in AR has already been shown to be more effective than a standard ultrasound screen as it avoids the user having to divert their eyes from the area of interest [5]. Another study showed that AR technology can reduce the perceived mental workload when inserting intravenous catheters [6]. Yet more studies showed that AR can be of benefit in nerve block procedures and to help guide biopsies [7, 8]. Furthermore, the use of AR technology has been shown to reduce complications when performing ultrasound guided-techniques [9]. 

Currently, one popular method of allowing the detection is to use an ultrasound probe with an QR code fixed to it, allowing the researchers to display the AR image on it [10, 11]. Other methods include using markers affixed to the probe which are detected by a set of cameras which allow for the placement of the image over the ultrasound probe [12]. 

Interestingly, the HoloLens (Microsoft, USA) platform has emerged as a particularly as one of the main ways that AR technology has been paired with ultrasound, likely due to the ability to create applications which can be used to display the images. However, price has always been a drawback as mentioned by multiple studies, which often used AR and VR headsets costing around 800 USD or others which used material that cost substantially more [5, 6]. 

Hand-tracking technology is highly complex but has developed from only being able to be deployed on expensive systems to slowly being used in commercial devices [13]. Though previously requiring the use of extra materials, such as colouring each individual finger segment, in order to properly detect each digit, the technology has now improved to the point where hand tracking is able to be deployed by relatively inexpensive consumer devices [13, 14]. Hand tracking has become particularly important as it allows for more immersive interaction that is possible with controllers and also allows for interaction with objects in a more natural fashion [14]. In the medical field, the development of hand-tracking technology has allowed for the possibility of working in a sterile manner, all the while being able to manipulate the ultrasound settings in AR without the need for additional help.

We present a novel way of using AR technology in combination with ultrasound and explain how to do it. Using this technique, it is possible to perform an ultrasound all while remaining sterile and manipulating the interface via augmented reality.

## Methods

The material that we used was a Meta Quest 3 (Meta, USA) headset, due to its ability to perform hand tracking in AR, and the fact that it works using an Android operating system. We also used a Lumify L 12 − 4 and C 5 − 2 probe (Phillips, Netherlands), but have subsequently attempted the procedure with a 3D probe (Sureresult, China), with both being able to work perfectly. We therefore believe that it should be possible to replicate this procedure with any portable ultrasound probe that can be linked to an Android app that is able to be sideloaded onto the headset.

The first step is to put the Quest 3 into developer mode to allow for the installation of the necessary application. This can be done using the Meta Horizon app, with instructions on how to do so easily accessible online.

The next step is to download the Android application app for the probe that you are using, such as Philips Lumify Ultrasound App. As they are not included in the Meta app store, they must be uploaded to the Quest 3 using the “APK” (application) file. These files can be easily found and downloaded from the internet.

To use the app onto the Quest 3, one must use a procedure known as “sideloading” which is done to allow the user to upload Android apps which are not available on the Meta store. To do this we used the “SideQuest” app (https://sidequestvr.com/) due to our pre-existing familiarity with it, though other apps exist. The instructions for how to upload APK files is available on their website and thus will not be explained here.

Once the application is uploaded to the headset, it must simply be accessed through the menu and can then be manipulated using hand tracking, making the need for touching a physical ultrasound machine obsolete. At the time of writing, due to the app not being acquired from Meta’s app store, it will appear under “unknown sources” within the Quest’s menu. However, once it has been opened it will subsequently appear within the toolbar located at the bottom of the menu to allow for easy access until it is replaced by opening more apps.

Once it has been opened, the app screen can be resized and moved around at will using the hand-tracking. This allows the operator full control over where to place the screen in their environment.

**Fig. 1:**
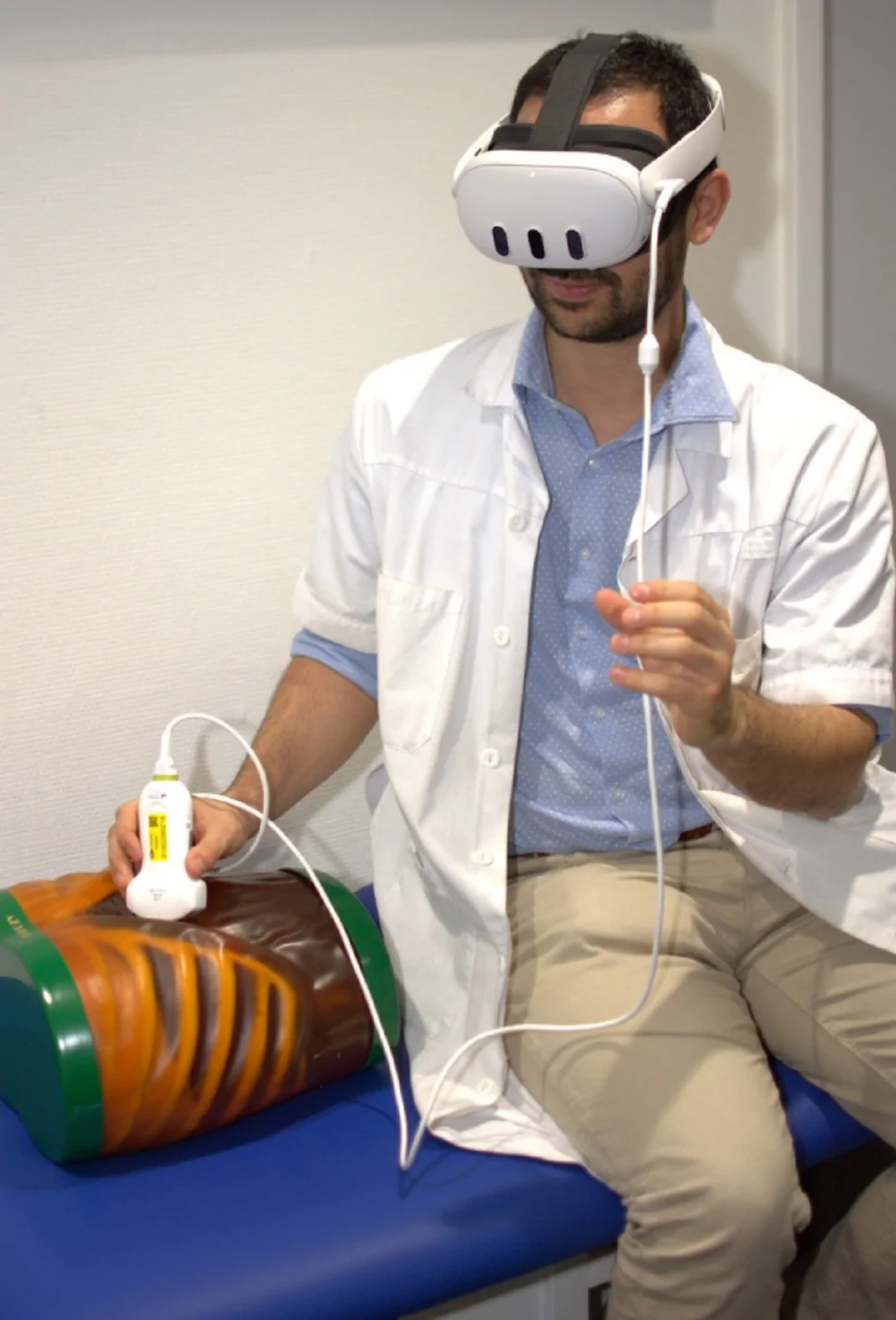
Image showing the material needed to perform the examination

The ultrasound probe can then either be plugged into the headset using a USB-C connector or can be connected over Wi-Fi, depending on the model of ultrasound probe being used. Once it has been recognised and accepted, the probe should automatically activate and can begin to be used. It should be noted that some probes may require the users to sign-in to use the app and therefore an internet connection may be required (Fig. 1).

Axial and lateral resolution were assessed using the combined resolution target groups at a depth of 3 cm for the L12-4 probe and 10.5 cm for the C5-2 probe. Contrast resolution for low-contrast structures was evaluated by determining the smallest visible diameter of the anechoic stepped cylinders at a depth of 1.5 cm for the L12-4 probe and 11.5 cm for the C5-2 probe. Grayscale contrast sensitivity was assessed using grayscale target groups ranging from − 9 dB to > 15 dB relative to the background at 3 cm (six targets) for the L12-4 probe, and from − 6 dB to > 15 dB at 11.5 cm (five targets) for the C5-2 probe.

Manipulation of the application was performed with bare hands, flesh-colored sterile gloves (Cardinal Health Surgical Gloves, Protexis Latex Micro), or blue sterile undergloves (Molnlycke Biogel PI Indicator synthetic non-natural rubber latex) and green sterile undergloves (Molnlycke Biogel Eclipse Indicator natural rubber latex), to qualitatively assess differences in hand tracking. This was done following a predefined sequence: adjusting the app window (closer, farther, right slot, central slot), selecting preset, setting gain and depth (minimal, maximal, ideal), freezing, measuring, and unfreezing, using color Doppler (high flow, box centering, low flow), Doppler pulse with gate positions (top left, top right, bottom right, bottom left, center) and angle correction (-60° to + 60°), activating spectral mode, and performing a final freeze.

In addition, the capability of connecting the HMD to Reacts (Philips Tele-Ultrasound Platform) was tested focusing on qualitative assessment of microphone, video, and ultrasound image sharing. (link: https://www.itnonline.com/content/philips-integrates-reacts-tele-ultrasound-platform-lumify-portable-systems)

## Results

**Fig. 2:**
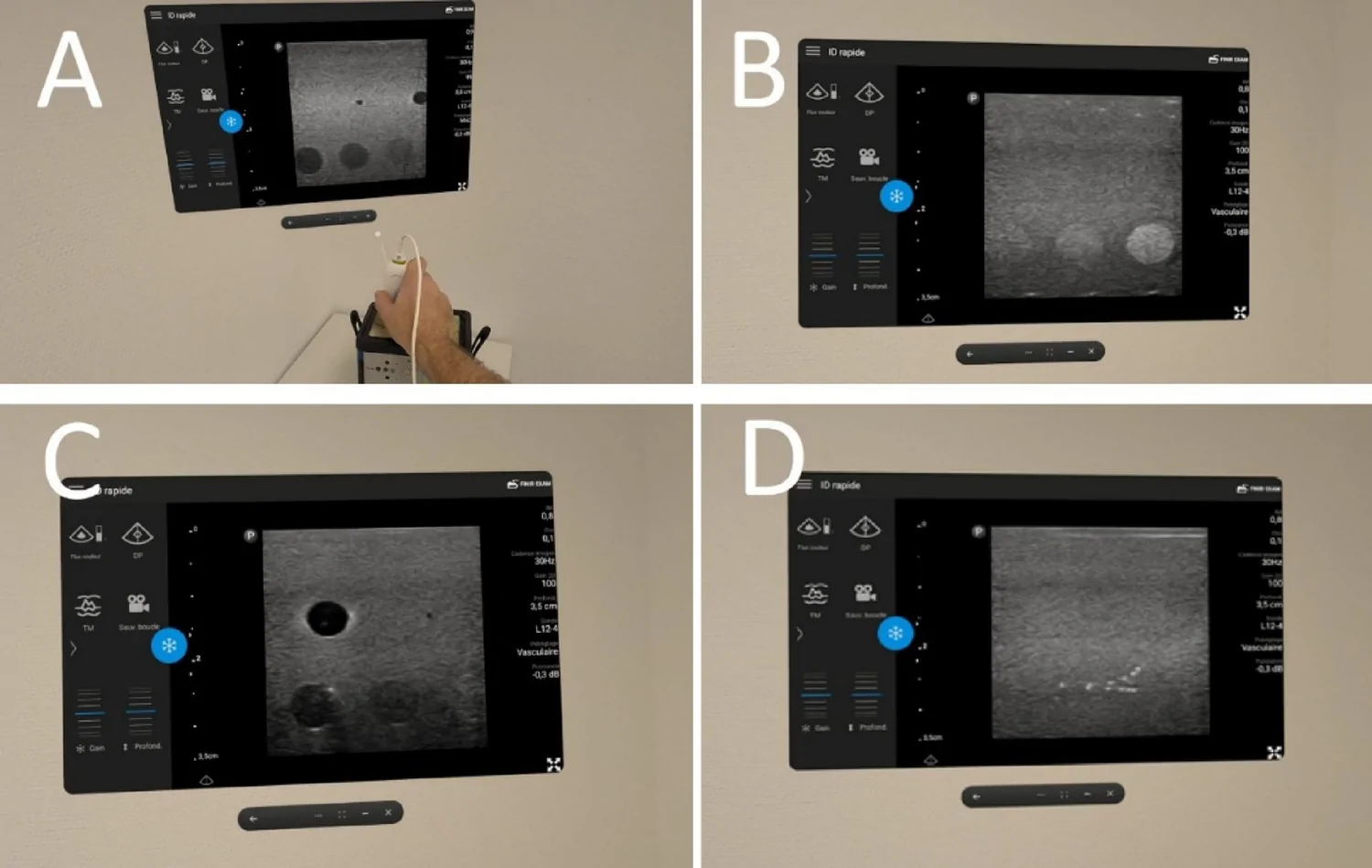
Images (A-D) taken using the headset showing the different cylinders of the Multi-Purpose Multi-Tissue Ultrasound Phantom (CIRS Model 040GSE) as seen through the Meta Quest 3

As the Quest 3 uses an Android operating system, the apps have full functionality and can be used as they would be on a tablet.

The entire setup, including the installation of the APK on the headset can be reasonably achieved in 30 min, with the subsequent use of the probe alongside the headset requiring only the time it takes to turn on the headset and plug in the probe. This methodology also does not require any technical skills and should be accessible to the vast majority of people who have access to both an AR headset running an android-based operating system and a portable ultrasound machine which works using android apps.

Although we have only tried our technique on the Meta Quest 3 headset, we believe that it should be possible to achieve similar results using any AR headset that uses android and is capable of hand-tracking. However, the steps needed to upload the APK file may vary on other devices.

Imaging performance was examined in a semi-quantitative way by comparing between images generated by the probe. Testing was performed using a Multi-Purpose Multi-Tissue Ultrasound Phantom (CIRS Model 040GSE), which enables semi-quantitative evaluation of imaging performance (Fig. 2). To ensure controlled display conditions, and because importing images into the Lumify^®^ app was not possible, we compared the same static image exported from the HMD in DICOM RLE (Run-Length Encoding) format and viewed in a locally running DICOM viewer (https://play.google.com/store/apps/details? id=com.posko777.tool.dicomviewer&pcampaignid=web_share) on both the HMD and the tablet (Samsung T830).

**Fig. 3:**
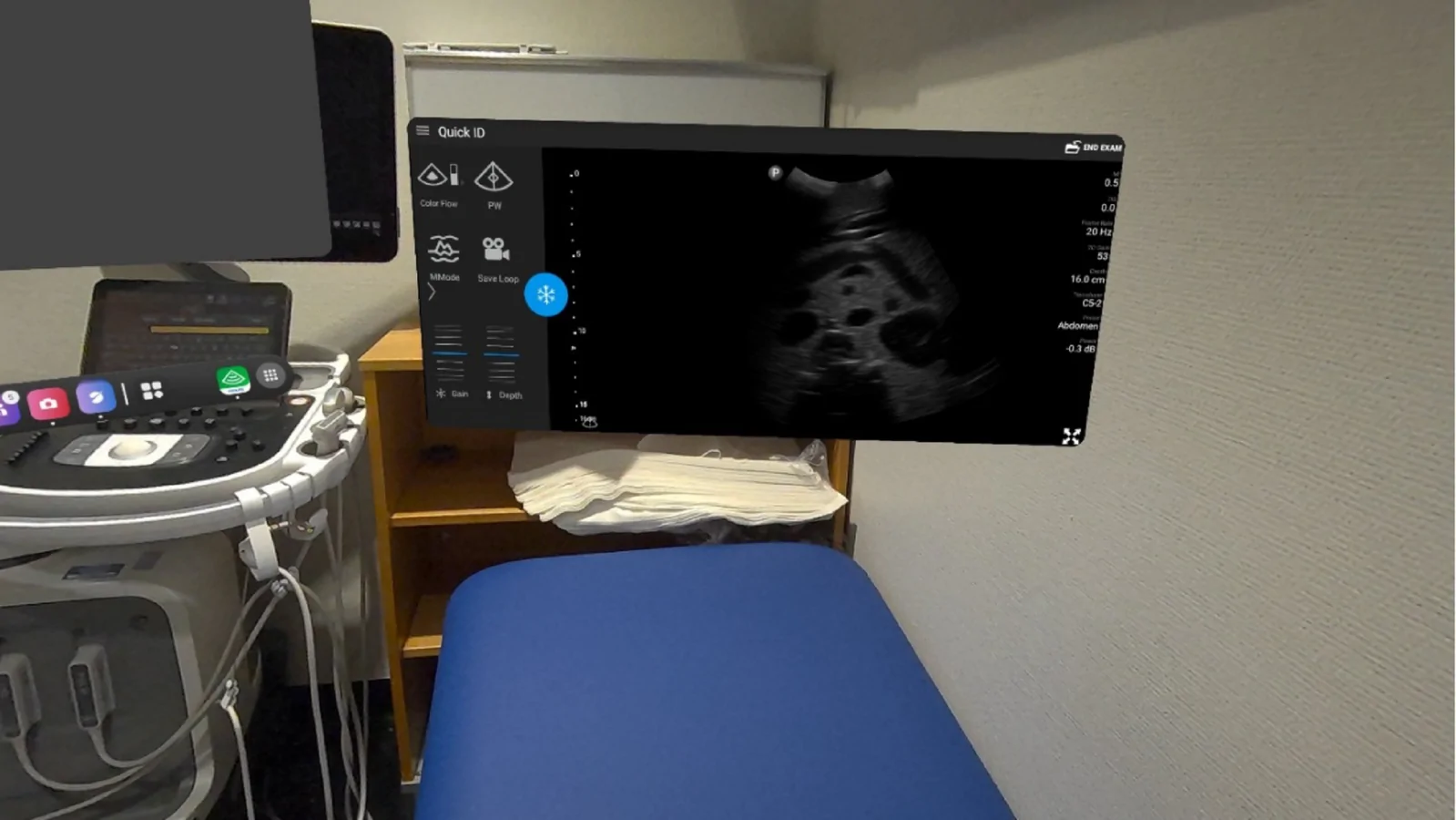
Still image taken using the headset showing the quality of the ultrasound image that can be acquired (image acquired using the Lumify C 5 − 2 probe on an abdomen ultrasound phantom)

**Fig. 4:**
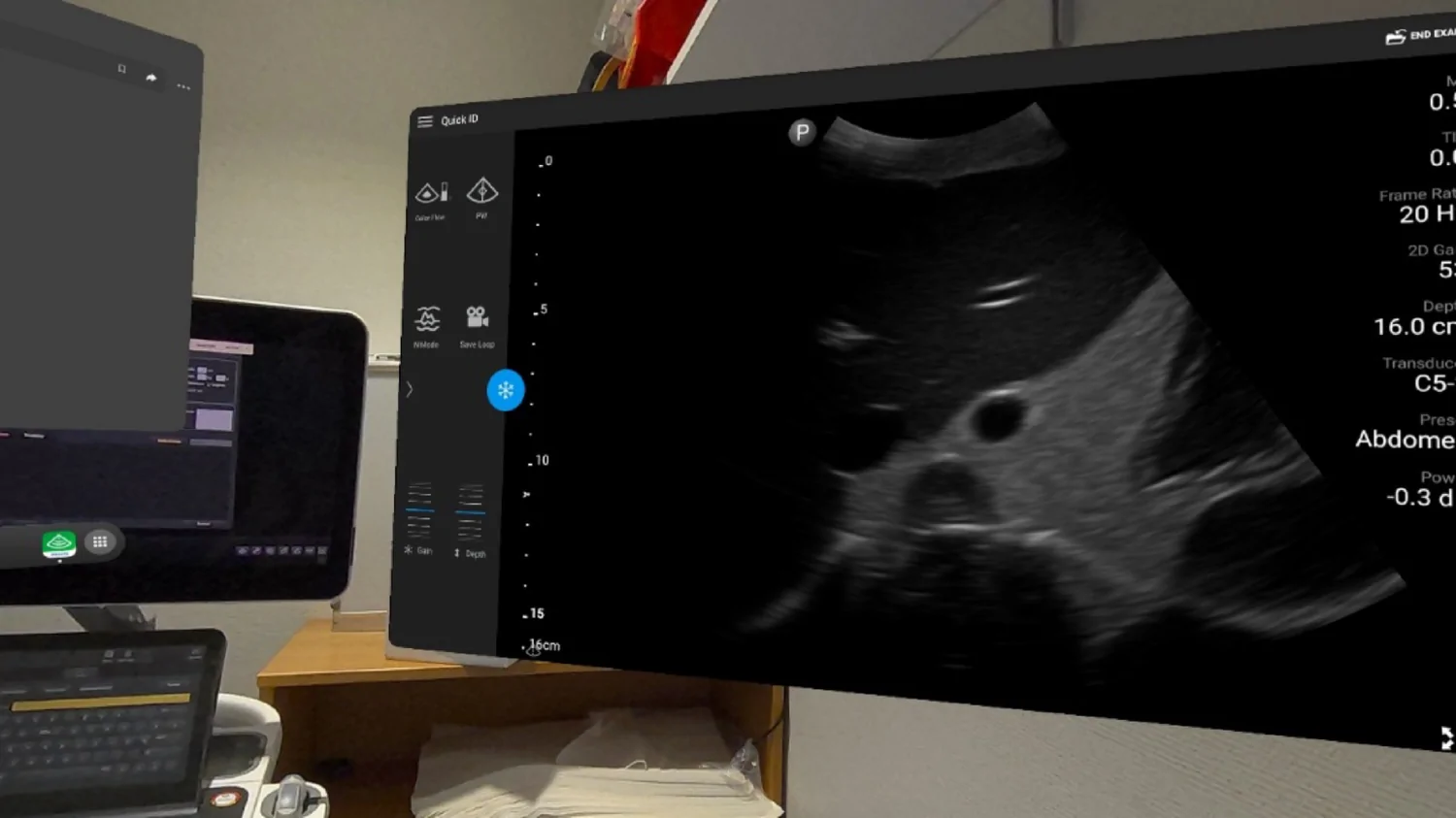
Still image taken using the headset showing a re-sized image to a larger format that can be placed in proximity to the patient (image acquired using the Lumify C 5 − 2 probe on an abdomen ultrasound phantom)

We did not find that the quality of the image was any less clear than on a tablet (Figs. 3 and 4, and Table 1).), owing to the equivalent of a 4 K display for each eye [16]. The comparison was possible as the images visualised on both the tablet and Quest 3 were identical, allowing their direct comparison. Furthermore, as the ultrasound image is directly projected into the headset, which blocks out any external light, there is the extra benefit of the image not being susceptible to reflections off the screen and thus the problem of glare is eliminated. This is particularly advantageous in brightly lit environments, such as are often encountered when working in hospital settings.

**Fig. 5:**
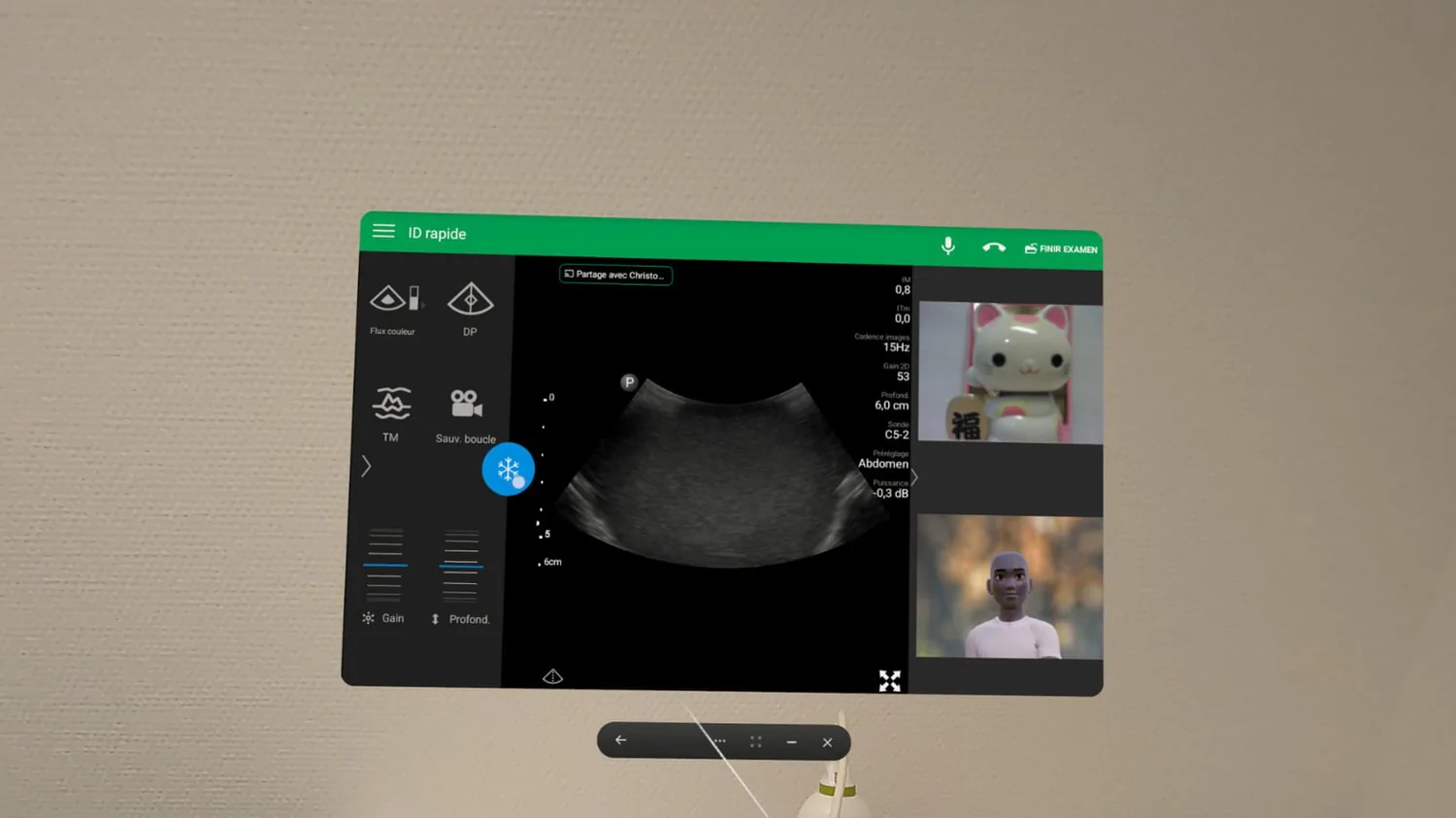
Image showing the use of the Reacts (the Philips Telehealth platform), showing the feasibility of transmitting images in real-time. In the standard configuration the HMD user’s camera is replaced by the person’s Meta avatar in the bottom right image. The person being called has their camera’s video feed being shown in the top right (Maneki-neko cat image).

**Table 1:** Table showing the semi-quantitative assessment between the tablet (Samsung T830) and the Meta Quest 3

Probe	CIRS 040	Depth (cm)	Tablet	Meta Quest 3	
L12-4	Axial Resolution (mm)	3	0.5	0.5	
L12-4	Lateral Resolution (mm)	3	1	1	
L12-4	Low-contrast detectability (mm)*	1.5	1.3	1.3	
L12-4	Gray Scale Contrast Sensitivity (number of visible targets)**	3	6/6 (100%)	6/6 (100%)	
C5-2	Axial Resolution (mm)	10.5	1	1	
C5-2	Lateral Resolution (mm)	10.5	> 5	> 5	
C5-2	Low-contrast detectability (mm)*	10	3	3	
C5-2	Gray Scale Contrast Sensitivity (number of visible targets)**	11.5	5/5 (100%)	5/5 (100%)	

* : Anechoic targets at 1.5 cm depth are stepped at 1.3, 2, 3, 4.5, and 6.7 mm, while anechoic targets at 10 cm depth are stepped at 2,3,4.5,6.7, and 10 mm

** : Targets at 3 cm depth have contrast levels of − 9, −6, − 3, +3, + 6, and > + 15 dB, while targets at 11.5 cm depth have contrast levels of − 6, −3, + 3, +6, and > + 15 dB, relative to the background

The use of surgical gloves did not appear to impact the hand tracking in a significant way, reinforcing our certainty that this can be used in to perform sterile procedures (Video 1, 2, and3). This means that there is no loss of functionality and that the probe can be used to its full potential, as it would be with a tablet.

The Reacts system was tested and functioned properly; within the HMD configuration, the tablet’s front camera filming the operator was replaced with a video of the Meta3 avatar. (Table 2; Fig. 5)

**Table 2:** Table showing the qualitative appreciation of hand tracking with bare hands and whilst wearing a series of different gloves as well as the use of the Reacts application

HMD	Sequence completion	Qualitative appreciation
Hand Tracking with Bare hands	Yes	Adequate
Hand Tracking with Flesh colored sterile gloves	Yes	Adequate
Hand Tracking with Blue colored sterile undergloves	Yes	Adequate
Hand Tracking with Green colored sterile undergloves	Yes	Adequate
REACTS ultrasound image sharing	Yes (without app adjustment and preset selection)	Adequate
REACTS video sharing	N/A	Adequate
REACTS microphone sharing	N/A	Adequate

Sequence: adjusting the app window (closer, farther, right slot, central slot), selecting preset, setting gain and depth (minimal, maximal, ideal), freezing, measuring, and unfreezing, using color Doppler (high flow, box centering, low flow), Doppler pulse with gate positions (top left, top right, bottom right, bottom left, center) and angle correction (-60° to + 60°), activating spectral mode, and performing a final freeze

N/A: not applicable

## Discussion

AR and VR technology are becoming increasingly used together with ultrasound for education as well as in applications such as vascular access, amongst others [5, 6, 9, 17–21]. 

Though widespread adoption remains relatively limited, point-of-care ultrasound (POCUS) is quickly growing and becoming a part of internal medicine training due to its ability to improve a clinician’s diagnosis [22]. 

Studies have shown that the portability of the POCUS devices significantly influences the likelihood that they will be used by clinicians by lowering the barriers to use which are present with more traditional cart-based ultrasound stations [23]. It therefore follows that better portability and ergonomics, such as is achievable with a VR headset, could be of benefit.

Previous recent studies have been conducted that used AR headsets coupled with ultrasound devices. One study used a Microsoft HoloLens 2 (Microsoft, USA) headset that received a portable ultrasound machine coupled to a tablet running a Windows (Mircrosoft, USA) operating system (OS), which then streamed the images to the headset in real time via WIFI [24]. However, this setup did not allow for the direct manipulation of the images and only allowed for the visualisation. Another study was performed using a portable computer, also running a Windows OS, as the intermediary before streaming to a HoloLens 2 [25]. This setup, however, did allow for the limited manipulation of the AR projection by moving and changing the size of the projected image.

Other studies have also shown the usefulness of technology that has a head-mounted display in performing many ultrasound-guided procedures, though the technology used only allowed for projecting the image without any forms of manipulation. One of these procedures included vascular access in an operation room setting, which requires total sterility [19]. In this example the researchers also used a fully wireless probe, which we have shown can also be done using our methodology, thus providing proof for a practical real-world use [19]. Further examples exist of similar procedures using other head-mounted displays for ultrasound-guided procedures [26]. However, in these cases simply represent the use of a head-up display and not true AR technology. Nevertheless, they provide true use-cases wherein displaying the images directly within the field of view has been shown to be useful.

Our methodology improves on the previous studies by allow complete manipulation of the screen location and size as well as, arguably more importantly, the ultrasound probe’s settings. This allows for complete control and for the execution of the ultrasound exam in a nearly identical way when compared to the usual tablet-based setup, without any loss of functionality as the Lumify app is used. Furthermore, by directly connecting ultrasound probe to the headset, our technique is more portable and removes possible connectivity issues related to having an intermediary system. Finally, the hardware that we used achieves these results at a fraction of the cost of the material used in the previous studies, making it far more financially accessible.

We also showed that we are able to manipulate the Lumify app using the hand tracking in conjunction with gloves. This can be particularly advantageous if operating a wireless ultrasound probe as it may allow for near-complete sterility by being able to prevent the need to touch any non-sterile material. As the application is the same as would be used on the tablet, all of the probe settings and functionalities (such as Doppler, gain, depth etc.) are fully accessible. This may potentially allow for the use of the ultrasound prove in environments that could have been problematic due to sterility issues, whilst giving the operator full control over the probe, reducing reliance on third parties and allowing full autonomy.

In areas where professionals collaborate using remote session, the possibility of sharing a live video feed from the headset, using technology such as Reacts (Phillips, Amsterdam, Netherlands) was also demonstrated as feasible. We also believe that using a non-proprietary video sharing could be possible.

Further evidence for the usefulness of AR ultrasound systems comes from studies showing that they can reduce the mental load when performing vascular access procedures [6]. This can be useful in helping younger physicians, particularly when first learning to use ultrasound. In these cases, our methodology can also provide extra benefits since the Quest 3 allows for multiple applications can be used in parallel. This means that the person using the headset can have videos, web pages or digital textbooks open in parallel which can be used to guide their exams or check their images against pathological examples. This could be particularly useful for students.

The complete control of the ultrasound system without the need for physical buttons is a great improvement over current methods as it reduces the need to handle an ultrasound computer and trolley. This is important both of which have been shown to often be contaminated by bacteria, even in ICU settings of high-income countries [15]. As such, this methodology might also be beneficial in reducing patient contamination in high-risk settings where asepsis must be maintained.

Currently, the main limitation of this technique is, in our view, the image quality of the portable ultrasound probes, which is not equivalent to cart-based ultrasound machines. However, if manufacturers see the value of this technology, we believe that it is technically feasible to control an ultrasound machine by duplicating the controls in way by which they can be accessed via an app. Currently, it should be noted that it is also possible to see the output of a “classic” ultrasound machine by wiring in the HDMI output into the Quest 3 using a HDMI-USB-C converter, though in that case one can only see the monitor’s replicated screen and, whilst it is possible to move it around and change its size, one must adjust the settings on the ultrasound machine as it is not possible to do so via the AR image.

## Conclusion

We present a novel way of using commercially available AR headset for viewing images from a portable ultrasound probe. This is the first time that the hand-tracking technology, included in the AR headset, is used in conjunction with the probe’s software to allow for hands-free manipulation of the probe’s settings, obviating the need for touching a tablet or the knobs of an ultrasound machine. Importantly, this method improves over existing ways of using AR in combination with ultrasound by being technically very simple to set up, opening the door to wide-ranging use of this technology.

## Supplementary Information

Below is the link to the electronic supplementary material.

Supplementary Material 1 Video 1:Video taken using the headset showing the possibility of resizing the screen and interacting with image depth settings in augmented reality.

Supplementary Material 2 Video 2:Video taken using the headset showing the manipulation of the screen in order to move it around.

Supplementary Material 3 Video 3:Demonstration of the hand-tracking being maintained whilst wearing sterile gloves and the use of various settings.

## Data Availability

No data was generated during the creation of this manuscript.

## References

[1] Saliba T, Boitsios G, Preziosi M, Negro G, De Leucio A, Simoni P. Virtual reality simulations to alleviate fear and anxiety in children awaiting MRI: a small-scale randomized controlled trial. J Clin Monit Comput [Internet] Springer Sci Bus Media B V. 2024;1–10. 10.1007/S10877-024-01188-5/TABLES/4. [cited 2024 Aug 21];. 38954169

[2] Antonovics E, Boitsios G, Saliba T. Use of virtual reality in children in a broad range of medical settings: a systematic narrative review of recent meta-analyses. Clin Exp Pediatr [Internet] Korean Pediatr Soc. 2024;67:274–82. 10.3345/CEP.2023.00388. [cited 2024 Aug 21];. PMC11150988 38772412

[3] Gullo G, Rotzinger DC, Colin A, Frossard P, Gudmundsson L, Jouannic AM, et al. Virtually Augmented Self-Hypnosis in Peripheral Vascular Intervention: A Randomized Controlled Trial. Cardiovasc Intervent Radiol Springer. 2023;46:786–93. 10.1007/s00270-023-03394-1. PMC10030078 36944851

[4] Fuchs H, State A, Pisano ED, Garrett WF, Hirota G, Livingston M, et al. Towards performing ultrasound-guided needle biopsies from within a head-mounted display. Lecture Notes in Computer Science (including subseries Lecture Notes in Artificial Intelligence and Lecture Notes in Bioinformatics) [Internet]. Volume 1131. Berlin, Heidelberg: Springer; 1996. pp. 591–600. [cited 2025 Sep 28];. 10.1007/BFB0047002.

[5] Jang YE, Cho SA, Ji SH, Kim EH, Lee JH, Kim HS, et al. Smart Glasses for Radial Arterial Catheterization in Pediatric Patients: A Randomized Clinical Trial. Anesthesiology [Internet]. Volume 135. Lippincott Williams and Wilkins; 2021. pp. 612–20. [cited 2025 Jan 26];. 10.1097/ALN.0000000000003914. 34352073

[6] Liao SC, Shao SC, Gao SY, Lai ECC. Augmented reality visualization for ultrasound-guided interventions: a pilot randomized crossover trial to assess trainee performance and cognitive load. BMC Med Educ. 2024;24:1058. 10.1186/s12909-024-05998-8. PMC11429828 39334275

[7] Kasuya Y, Moriwaki S, Inano C, Fukada T, Komatsu R, Ozaki M. Feasibility of the head-mounted display for ultrasound-guided nerve blocks: a pilot simulator study. J Anesth [Internet]. 2017;31:782–4. 10.1007/S00540-017-2371-X/TABLES/1. [cited 2025 Jan 26];. Springer Tokyo. 28477229

[8] Shimizu T, Oba T, Ito KI. The Advantage of Using an Optical See-Through Head-Mounted Display in Ultrasonography-Guided Needle Biopsy Procedures: A Prospective Randomized Study. J Clin Med [Internet]. 2023;12:512. 10.3390/JCM12020512/S1. [cited 2025 Jan 26];. PMC9865023 36675443

[9] Rüger C, Feufel MA, Moosburner S, Özbek C, Pratschke J, Sauer IM. Ultrasound in augmented reality: a mixed-methods evaluation of head-mounted displays in image-guided interventions. Int J Comput Assist Radiol Surg Springer Sci Bus Media Deutschland GmbH. 2020;15:1895–905. 10.1007/s11548-020-02236-6. PMC8332636 32725398

[10] Costa N, Ferreira L, de Araújo ARVF, Oliveira B, Torres HR, Morais P, et al. Augmented Reality-Assisted Ultrasound Breast Biopsy. Sens MDPI. 2023;23. 10.3390/s23041838. PMC9961993 36850436

[11] Farshad-Amacker NA, Kubik-Huch RA, Kolling C, Leo C, Goldhahn J. Learning how to perform ultrasound-guided interventions with and without augmented reality visualization: a randomized study. Eur Radiol [Internet]. Springer Science and Business Media Deutschland GmbH; 2023 [cited 2024 Apr 20];33:2927–34. 10.1007/S00330-022-09220-5/FIGURES/6 PMC10017581 36350392

[12] von Haxthausen F, Moreta-Martinez R, de la Pose Díez A, Pascau J, Ernst F. UltrARsound: in situ visualization of live ultrasound images using HoloLens 2. Int J Comput Assist Radiol Surg Springer Sci Bus Media Deutschland GmbH. 2022;17:2081–91. 10.1007/s11548-022-02695-z. PMC9515035 35776399

[13] Wang RY, Popović J. Real-time hand-tracking with a color glove. ACM Trans Graph. 2009. 10.1145/1531326.1531369.

[14] Buckingham G. Hand Tracking for Immersive Virtual Reality: Opportunities and Challenges. Front Virtual Real. Front Media S A. 2021;2. 10.3389/frvir.2021.728461.

[15] Meta Quest 3 128GB [Internet]. [cited 2024 Mar 17]. Available from: https://unboundxr.eu/meta-quest-3-128gb

[16] Li H, Yan W, Zhao J, Ji Y, Qian L, Ding H et al. Navigate biopsy with ultrasound under augmented reality device: Towards higher system performance. Comput Biol Med. Elsevier Ltd; 2024;174. 10.1016/j.compbiomed.2024.108453 38636327

[17] Saruwatari MS, Nguyen TN, Talari HF, Matisoff AJ, Sharma KV, Donoho KG, et al. Assessing the Effect of Augmented Reality on Procedural Outcomes During Ultrasound-Guided Vascular Access. Ultrasound Med Biol Elsevier Inc. 2023;49:2346–53. 10.1016/j.ultrasmedbio.2023.07.011. PMC10658651 37573178

[18] Al-Abcha A, Alkhouli M, Prasad A, Bell M, Yalamuri S, Lerman A, et al. Augmented Reality and Ultrasound-Guided Vascular Access. Circ Cardiovasc Interv Lippincott Williams Wilkins. 2023;16:E013360. 10.1161/CIRCINTERVENTIONS.123.013360. 37650286

[19] Marhofer P, Eichenberger U. Augmented reality in ultrasound-guided regional anaesthesia: useful tool or expensive toy? Br J Anaesth Elsevier. 2023;131:442–5. 10.1016/J.BJA.2023.05.022. 37353469

[20] Saliba T, Pather S. The use of virtual reality and augmented reality in ultrasound education, a narrative review of the literature. Journal of Clinical Ultrasound [Internet]. John Wiley & Sons, Ltd; 2024 [cited 2024 Dec 8]; 10.1002/JCU.23840 39304523

[21] Yamada T, Soni NJ, Minami T, Kitano Y, Yoshino S, Mabuchi S et al. Facilitators, barriers, and changes in POCUS use: longitudinal follow-up after participation in a national point-of-care ultrasound training course in Japan. Ultrasound Journal. Springer-Verlag Italia s.r.l.; 2024;16. 10.1186/s13089-024-00384-3 PMC11231124 38976114

[22] Glogoza M, Urbach J, Rosborough TK, Olet S, St. Hill CA, Smith CS, et al. Tablet vs. station-based laptop ultrasound devices increases internal medicine resident point-of-care ultrasound performance: a prospective cohort study. Ultrasound J Springer. 2020;12. 10.1186/s13089-020-00165-8. PMC7163000 32300979

[23] Zhang G, Nguyen TN, Fooladi-Talari H, Salvador T, Thomas K, Crowley D, et al. Augmented reality for point-of-care ultrasound-guided vascular access in pediatric patients using Microsoft HoloLens 2: a preliminary evaluation. J Med Imaging SPIE-Intl Soc Opt Eng. 2024;11. 10.1117/1.jmi.11.6.062604. PMC11393663 39280781

[24] Evans KD, Bradbury P, Bloom IW, Xu M. A Product Evaluation of Augmented Reality Equipment Coupled to Diagnostic Medical Sonography: A Potential Equipment and Ergonomic Innovation. Journal of Diagnostic Medical Sonography. Volume 41. SAGE Publications Inc.; 2025. pp. 57–64. 10.1177/87564793241268415.

[25] Wu TS, Dameff CJ, Tully JL. Ultrasound-guided central venous access using google glass. J Emerg Med Elsevier Inc. 2014;47:668–75. 10.1016/j.jemermed.2014.07.045. 25281180

[26] Heldeweg MLA, Berend K, Cadenau L, Rosingh A, Duits AJ, van Mansfeld R, et al. Bacterial Contamination of Ultrasound and Stethoscope Surfaces in Low- and High-Resource Settings. Am J Trop Med Hygiene Am Soc Trop Med Hygiene. 2022;107:463–6. 10.4269/ajtmh.22-0074. PMC9393444 35895395

